# Constrictive Pericarditis: A Commonly Missed Cause of Treatable Diastolic Heart Failure

**DOI:** 10.7759/cureus.8024

**Published:** 2020-05-08

**Authors:** Pradnya Brijmohan Bhattad, Vinay Jain

**Affiliations:** 1 Internal Medicine, East Tennessee State University, Johnson City, USA; 2 Radiology, James H. Quillen Veterans Affairs Medical Center, Johnson City, USA

**Keywords:** constrictive pericarditis, diastolic heart failure

## Abstract

Constrictive pericarditis arises as a result of the fibrous thickening of the pericardium due to chronic inflammatory changes from various injuries. Increased pulmonary and systemic venous pressures manifest clinical features of left and right heart failure. Idiopathic or post-viral pericarditis is the most common cause followed by postpericardiotomy, radiation-induced causes. Right-sided heart failure symptoms predominate over left-sided heart failure symptoms due to the equalization of pressures. No single diagnostic test can provide a definitive diagnosis or evidence of constrictive pericarditis. Medical management is difficult for constrictive pericarditis. The treatment of choice for constrictive pericarditis is pericardiectomy.

## Introduction and background

Chronic inflammation leads to pericardial scarring and fibrous thickening, which causes constrictive pericarditis [1-2]. The obliteration of pericardial space with loss of normal pericardial compliance interferes with cardiac filling. There is a dissociation of intracardiac pressure from intrathoracic pressure. A characteristic feature of constrictive pericarditis is the equalization of end-diastolic pressures in all four cardiac chambers [2-5]. Individuals with constrictive pericarditis exhibit features of both right and left heart failure. Constrictive pericarditis is a rare cause of heart failure, with increasing prevalence, and the diagnosis is often missed [2-3,5-6].

## Review

Etiology

Chronic pericardial inflammation can result in constrictive pericarditis. Tuberculosis is the most common cause of constrictive pericarditis in developing regions of the world [6,7-9]. The incidence of constrictive pericarditis secondary to tuberculosis has dropped precipitously in the United States due to effective antitubercular medications. Bacterial infections are another cause of constrictive pericarditis in the developing world but there is a very low number of cases in the United States from bacterial causes due to antibiotics or drainage procedures [5,8-11]. Commonly, idiopathic cases of constrictive pericarditis are likely infectious in etiology, secondary to viral infections by viruses such as coxsackievirus and echovirus. Fungal and parasitic agents are less common causes of infectious etiology [7-8,10]. Constrictive pericarditis as a late complication of radiation therapy usually occurs several years after radiation therapy. Radiation-induced constrictive pericarditis may have associated radiation damage to the myocardium contrary to other causes of constrictive pericarditis where the myocardium is structurally and functionally normal [8-9,11]. Risk factors leading to post-cardiac surgery constrictive pericarditis are intraoperative hemorrhage into the pericardium, postpericardiotomy syndrome, and postoperative pericarditis. It is a late complication of cardiac surgery such as valvular surgery or coronary artery bypass graft. Neoplastic diseases, mainly breast cancer, lung cancer, lymphoma, mesothelioma, and melanoma, are uncommon causes. Idiopathic or post-viral pericarditis is the most common cause followed by postpericardiotomy, radiation-induced causes [9,12-13]. Refer to Table 1.

**Table 1:** Etiology of constrictive pericarditis

Common Causes of Constrictive Pericarditis
Idiopathic pericarditis
Viral pericarditis
Post pericardiotomy
Chest irradiation
End-stage renal disease
Collagen vascular disease
Tuberculosis
Malignancy
Infectious diseases
Trauma

Pathophysiology

Thickening and fibrous scarring with adhesions of the two pericardial layers leads to obliteration of the pericardial cavity and makes the pericardium rigid and thickened [10,12,14]. This results in decreased ventricular compliance and thus inhibits adequate diastolic ventricular filling leading to increased venous pressures with decreased stroke volume. Equalization of end-diastolic pressures in all four cardiac chambers then occurs and is a hallmark feature of constrictive pericarditis [13,15-16]. Systole is usually unimpaired, however, marked diastolic dysfunction occurs. Diastolic dysfunction in constrictive pericarditis leads to most of the ventricular filling in the second phase of diastole with minimum ventricular filling in the third and fourth phases of diastole [14,17-19].

Clinical presentation

Clinical features of constrictive pericarditis are due to left and right-sided heart failure and elevated filling pressures. Early symptoms include nonspecific features such as fatigue, malaise, and decreased exercise tolerance [16-17,19]. As the filling pressures keep increasing with the disease progression, symptoms suggestive of low cardiac output and systemic congestion occur. Marked jugular venous distention, peripheral edema, ascites, hepatomegaly, splenomegaly, worsening exercise tolerance, exertional dyspnea, orthopnea, paroxysmal nocturnal dyspnea, and cough are commonly seen. Right-sided heart failure symptoms predominate over left-sided heart failure symptoms due to the equalization of pressures [14,19-24].

Kussmaul’s sign (lack of inspiratory fall in jugular venous distention due to increased venous return) is commonly noted. Kussmaul’s sign is sensitive but lacks specificity. Increased jugular venous pulsation with a prominent y descent is produced by rapid early diastolic ventricular filling. Cardiac auscultation made muffled heart sounds, a soft first heart sound, and an occasional pericardial knock. A pericardial knock is heard in early diastole, 0.06 to 0.1 seconds after the second heart sound and it indicates abrupt cessation of diastolic filling [20,22,25]. Pericardial knock is a high frequency sound best heard with the diaphragm of the stethoscope. The jugular venous pressure is occasionally very high in constrictive pericarditis such that the level is not evident on examination of the patient at a 45° angle and hence the diagnosis is missed. The height of the jugular venous pressure may sometimes only be evident on examination of the patient upright [21-23,25].

Diagnostic testing

No single diagnostic test can provide a definitive diagnosis or evidence of constrictive pericarditis. Several different diagnostic tests may be sometimes obtained when there is a high clinical suspicion [23-24,26]. As no gold standard diagnostic test exists, making a definitive diagnosis is often challenging.

The evaluation of the diastolic flow pattern with Doppler and the respiratory changes in the Doppler diastolic flow patterns may assist in providing significant evidence of constrictive pericarditis [24-25,27]. Generally, both left and right heart catheterization are performed for obtaining simultaneous ventricular pressure readings. Preserved x descent and the steep y decent results in the classic W-shaped atrial waveform configuration that can be found on right atrial pressure waveforms. Hypovolemia can mask the features of constrictive pericarditis and thus the fluid challenge in the cardiac catheterization laboratory may be required to unmask ventricular interdependence in the patients who are volume-depleted [26,28-30].

Cardiac computed tomography (CT) and magnetic resonance imaging (MRI) are supportive and nondiagnostic for constrictive pericarditis. A thickened pericardium of more than 4 mm on cardiac CT is supportive of the diagnosis and the best modality for the evaluation of pericardial calcification. Cardiac MRI may show ventricular interdependence and septal bounce and is the best modality to evaluate pericardial inflammation. Cardiac CT and/or MRI are useful to evaluate the extent of pericardial thickening and plan for pericardiectomy [27,29,31]. Refer to Table 2.

**Table 2:** Diagnostic Testing in Constrictive Pericarditis

Diagnostic test	Characteristic findings in constrictive pericarditis
1. Chest radiograph	-Pericardial calcification, commonly involving the right ventricle and atrioventricular groove. Pleural effusions and evidence of left and right atrial enlargement.
2.Electrocardiogram	-Low voltage QRS complexes, generalized flattening or inversion of the T waves, left atrial enlargement, atrial fibrillation.
3. Two-dimensional echocardiography	-Thickened, echogenic pericardium; rapid early diastolic pressures; attenuated late diastolic filling; tram-tracking; posterior left ventricular wall flattening in diastole; biatrial enlargement. -Septal bounce which is the sudden cessation of septal motion due to rapid early diastolic filling. -Dilated and noncompressible inferior vena cava due to elevated right-sided pressures. -Ventricular interdependence wherein the preferential filling of the right ventricle on inspiration makes the septum to move to the left and the augmentation of left ventricular filling on expiration makes the septum to move to the right.
4. Doppler echocardiography	A decrease in the mitral flow during inspiration, or an increase in the tricuspid flow during inspiration, decreased tricuspid flow during expiration is noted. -The pulmonary systolic/diastolic flow ratio is decreased. -Systolic and diastolic pulmonary venous flows are markedly increased during expiration. -Doppler velocities of the median mitral valve annulus in early diastole are normal or slightly increased.
5. Cardiac catheterization	-Cardiac catheterization can demonstrative ventricular interdependence which is a hallmark of constrictive pericarditis. -Hemodynamic measurements remonstrate elevated and equal pressures in all four cardiac chambers in diastole. -Ventricular hemodynamics demonstrate the dip-and-plateau or square root sign during diastole. -Preserved x descent and steep y descent due to increased early diastolic flow can be noted on right atrial measurements.

Therapy

Medical Therapy

Medical management is difficult for constrictive pericarditis. Patients with New York Heart Association class I symptoms can be initially started on diuretics and a low sodium diet. Nonsteroidal anti-inflammatory drugs (NSAIDs), colchicine, and steroids may be beneficial in constrictive pericarditis, however, the majority of these patients usually require pericardiectomy [28,30-33]. Medical therapy is recommended in patients with severe comorbidities and limited life expectancy or with an unacceptably high risk for operative mortality [27,29,32].

Surgical Therapy

The treatment of choice for constrictive pericarditis is pericardiectomy (surgical stripping and removal of both layers of the constricting pericardium) with symptomatic improvement in over 90% of patients after the procedure [29-30,32]. The operative mortality risk of pericardiectomy ranges between 5% and 20%. Patients with constrictive pericarditis secondary to viral or idiopathic etiologies have better surgical outcomes as compared to patients with radiation-induced constrictive pericarditis [28,31-33]. Early surgical intervention is advocated, as constrictive pericarditis is a progressive disease, and patients with a poor preoperative functional class are at the highest risk for perioperative death [30,33].

## Conclusions

The clinical features of constrictive pericarditis are due to left and right-sided heart failure. Right-sided heart failure symptoms predominate over left-sided heart failure symptoms due to the equalization of pressures in all four cardiac chambers. It is often challenging to make a definitive diagnosis of constrictive pericarditis, as no gold standard diagnostic test exists. As a result, the diagnosis of constrictive pericarditis is commonly missed. The treatment of choice for constrictive pericarditis is pericardiectomy, which leads to symptomatic improvement in over 90% of patients after the procedure. It is imperative to recognize the varied presenting features of constrictive pericarditis early in the disease course in order to ensure timely management for preventing the progression of the disease and obtaining improved outcomes.
